# Structure–Function Decoupling: A Novel Perspective for Understanding the Radiation-Induced Brain Injury in Patients With Nasopharyngeal Carcinoma

**DOI:** 10.3389/fnins.2022.915164

**Published:** 2022-07-04

**Authors:** Ya-fei Kang, Rui-ting Chen, Hao Ding, Li Li, Jian-ming Gao, Li-zhi Liu, You-ming Zhang

**Affiliations:** ^1^Shaanxi Provincial Key Research Center of Child Mental and Behavioral Health, School of Psychology, Shaanxi Normal University, Xi’an, China; ^2^Department of Radiology, Xiangya Hospital, Central South University, Changsha, China; ^3^Department of Radiology, Affiliated Hospital of Guilin Medical University, Guilin, China; ^4^Department of Radiology, The First Affiliated Hospital of Guangxi Medical University, Nanning, China; ^5^State Key Laboratory of Oncology in South China, Collaborative Innovation Center for Cancer Medicine, Sun Yat-sen University Cancer Center, Guangzhou, China; ^6^State Key Laboratory of Oncology in South China, Department of Radiation Oncology, Collaborative Innovation Center for Cancer Medicine, Sun Yat-sen University Cancer Center, Guangzhou, China; ^7^National Clinical Research Center for Geriatric Diseases, Xiangya Hospital, Central South University, Changsha, China

**Keywords:** nasopharyngeal carcinoma, structure–function coupling, radiation encephalopathy, ReHo/VBM, individual prediction

## Abstract

Radiation-induced functional and structural brain alterations are well documented in patients with nasopharyngeal carcinoma (NPC), followed by radiotherapy (RT); however, alterations in structure–function coupling remain largely unknown. Herein, we aimed to assess radiation-induced structure–function decoupling and its importance in predicting radiation encephalopathy (RE). We included 62 patients with NPC (22 patients in the pre-RT cohort, 18 patients in the post-RT-RE_+ve_ cohort, and 22 patients in the post-RT-RE_–ve_ cohort). A metric of regional homogeneity (ReHo)/voxel-based morphometry (VBM) was used to detect radiation-induced structure–function decoupling, which was then used as a feature to construct a predictive model for RE. Compared with the pre-RT group, patients in the post-RT group (which included post-RT-RE_+ve_ and post-RT-RE_–ve_) showed higher ReHo/VBM coupling values in the substantia nigra (SN), the putamen, and the bilateral thalamus and lower values in the brain stem, the cerebellum, the bilateral medial temporal lobes (MTLs), the bilateral insula, the right precentral and postcentral gyri, the medial prefrontal cortex (MPFC), and the left inferior parietal lobule (IPL). In the post-RT group, negative correlations were observed between maximum dosage of RT (MDRT) to the ipsilateral temporal lobe and ReHo/VBM values in the ipsilateral middle temporal gyrus (MTG). Moreover, structure–function decoupling in the bilateral superior temporal gyrus (STG), the bilateral precentral and postcentral gyri, the paracentral lobules, the right precuneus and IPL, and the right MPFC exhibited excellent predictive performance (accuracy = 88.0%) in identifying patients likely to develop RE. These findings show that ReHo/VBM may be a novel effective imaging metric that reflects the neural mechanism underlying RE in patients with NPC.

## Introduction

Radiotherapy (RT) is the mainstay of treatment for non-metastatic nasopharyngeal carcinoma (NPC) because of its high sensitivity to ionizing radiation. However, high-dose radiation inevitably causes acute or delayed toxicity. As a delayed radiation-related complication, radiation-induced brain injury (RBI) has recently attracted attention for its serious neuropsychiatric symptoms, unclear pathogenesis, and insufficient investigations on individual prediction (Tang et al., 2012). Therefore, an in-depth understanding of RBI pathogenesis and the ability to perform individual predictions are of great clinical significance.

Recently, several studies have used non-invasive functional magnetic resonance imaging (fMRI) techniques to examine radiation-induced structural and functional alterations in gray matter (GM; Lin et al., 2017; Ding et al., 2018; Zhang et al., 2018, 2021). Several studies of voxel-based morphometry (VBM) and surface-based morphometry have reported radiation-induced cortical volume, cortical thickness, and surface area abnormalities in multiple brain regions, such as the bilateral temporal lobes, the precentral gyrus, and the inferior parietal lobule (IPL; Lv et al., 2014; Lin et al., 2017; Zhang et al., 2021). Similarly, more recent fMRI studies have also documented altered regional neuronal activity and functional connectivity (FC) in the intratemporal and extratemporal lobes (Ma et al., 2017; Qiu et al., 2018; Zhang et al., 2020a,^2021^; Zhao et al., 2021). However, on close inspection of the distribution of the affected brain regions in previous structural and functional studies, we observed an interesting phenomenon; the affected brain regions with radiation-induced structural abnormalities were not well matched with those revealed by studies in the functional domain. This phenomenon indicates that an investigation into the relationship between structural and functional profiles, followed by RT, may be a new direction to elucidate the neural mechanism of RBI.

The nature of the relationship between functional co-activation and brain anatomy is one of the fundamental issues in neuroscience (Gu et al., 2021). Some investigators believe that cerebral functions are shaped and constrained by the underlying neuroanatomical structure (Honey et al., 2009; Shen et al., 2012; Hermundstad et al., 2013; Wang et al., 2017). Specific molecular traits and cellular organization may be responsible for particular cerebral functions. For example, the selective increase in the expression of gene zif268 and the N-methyl-D-aspartate receptor in CA1 neurons of the hippocampus are tightly associated with fear memory retrieval and long-term memory consolidation (Hall et al., 2001; Wittenberg and Tsien, 2002). Moreover, specific cell differentiation of excitatory neuronal populations in layers 2 and 3 pyramidal neurons and layer 5 pyramidal tract neurons of the primary motor cortex is anatomical substrates for the global assessment of motor performance and reinforcement-based motor learning of skilled behaviors (Anderson et al., 2010; Levy et al., 2020). The advent of the fMRI technique has enabled *in vivo* investigations of the cerebral structure–function relationship in human populations and diseases. One such study showed that improved alignment between the functional signals and the architecture of the underlying white matter network is associated with increased cognitive flexibility (Medaglia et al., 2018). However, to the best of our knowledge, radiation-induced changes in the structure–function coupling have not been investigated in patients with NPC.

The machine learning approach can help to screen out classification-related information from the altered structure–function coupling throughout the whole brain after RT, thus enabling the prediction of temporal lobe necrosis [also named radiation encephalopathy (RE)] in the early stage. Predictive MRI biomarkers of RE could enable the stratification of patients with NPC for customized treatment and thus, help to improve disease control and survival. Therefore, we aimed to construct the structure–function coupling-based predictive model to predict RE in patients with NPC after RT and, thereby, enable clinicians to take preventive strategies (e.g., neuroprotective agents) (Yang et al., 2021) to stop or slow down the deterioration of RBI (Zhang et al., 2020c). Considering that the median interval from RT to RE was approximately 38–45 months (Zhou et al., 2013; Feng et al., 2018), to ensure the clinical significance of individualized prediction, the proposed predictive model for RE should be developed and validated based on MRI data within the latency of RE.

In this study, we first computed VBM and ReHo mapping for each patient with NPC. The ReHo/VBM metric was then established to measure the radiation-induced effect on the structure–function relationship. Subsequently, we applied a machine learning strategy to evaluate the prediction ability of the ReHo/VBM metric for RE. Given our previously reported divergent patterns of radiation-induced structural and functional alterations in the GM of patients with NPC (Zhang et al., 2018, 2020c,^2021^), we hypothesized that the ReHo/VBM metric will be an effective imaging metric that reflects the radiation-induced altered structure–function relationship in patients with NPC. Our objectives for performing this study were as follows: (1) to investigate the ReHo/VBM alterations in the normal-appearing GM in patients with NPC, followed by RT; and (2) to build a predictive model for identifying the patients with NPC who are likely to develop RE.

## Materials and Methods

### Participants

**FIGURE 1:**
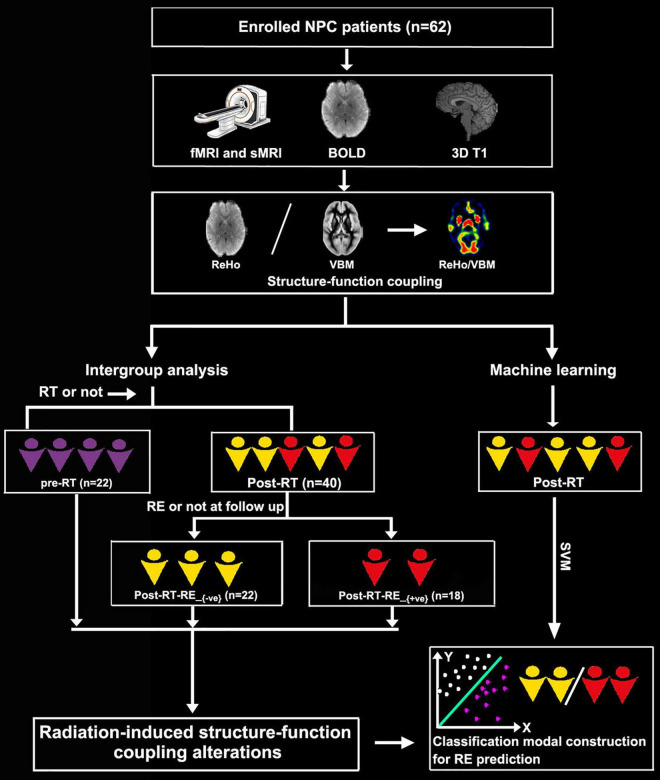
Flowchart for grouping and analysis of the enrolled patients with NPC. NPC, nasopharyngeal carcinoma; fMRI, functional MRI; sMRI, structural MRI; BOLD, blood oxygenation-level dependent; RT, radiotherapy; RE, radiation encephalopathy; ReHo, regional homogeneity; VBM, voxel-based morphometry.

We enrolled a total of 62 patients with NPC in this study, 22 of whom were in the pre-RT group, and the remaining 40 were in the post-RT group. According to the follow-up results of RE occurrence, the post-RT patients were further divided into two subgroups, namely, post-RT-RE_+ve_ (*n* = 18) and post-RT-RE_–ve_ (*n* = 22). The time intervals between RT and fMRI examinations were 10.77 ± 9.74 months in enrolled post-RT-RE_–ve_ patients with NPC and 17.00 ± 17.61 months in post-RT-RE_+ve_. The procedures for participant selection and grouping and the main analytical process are illustrated in Figure 1.

The clinical staging of patients with NPC was based on the seventh edition of the International Union against Cancer/American Joint Committee on Cancer (UICC/AJCC) TNM (T = Tumor, N = Nodes, and M = Metastasis) (2009). The post-RT patients were treated with intensity-modulated radiation therapy (IMRT) or two-dimensional conventional RT (2D-CRT); details are described in our previous work (Zhang et al., 2018). Concurrent chemoradiotherapy with/without neoadjuvant and/or adjuvant chemotherapy was recommended for patients at stages IIb to IVa–b (Zhang et al., 2021), alongside one or more chemotherapeutics, such as cisplatin, paclitaxel, nedaplatin, and fluorouracil. To minimize the confounding effect of chemotherapy on ReHo/VBM changes, efforts were made to balance the clinical stages of the enrolled patients with NPC between groups (Table 1) and standardize between-group chemotherapy regimens (Zhang et al., 2018, 2021). The inclusion criteria were as follows: (1) pathological evidence of NPC; (2) normal-appearing brain parenchyma on MRI; (3) aged <65 years; (4) right-handedness; and (5) Karnofsky Performance Scale score of >80; the subjects would be excluded if they presented with (1) brain tumor or atrophy, (2) brain invasion, (3) MRI contraindications, (4) a history of neuropsychological disorders and intracranial surgery, and (5) other substantial intracranial diseases (Zhang et al., 2018, 2021).

**TABLE 1:** Clinical parameters.

Clinical features	Pre-RT group (*n* = 22)	Post-RT-RE_–ve_ group (*n* = 22)	Post-RT-RE_+ve_ group (*n* = 18)	*P* value
***Sex, n***				
Female	4	6	4	0.927
Male	18	16	14	
***Age (years), mean* ± *SD***	43.82 ± 8.40	43.91 ± 10.27	45.28 ± 9.63	0.867
***Clinical staging***				
I/II, n	8	5	6	0.592
III/IV, n	14	17	12	
***RT technology***				
IMRT, n	NA	19	12	0.138
2D-CRT, n	NA	3	6	
***Time intervals between RT and fMRI examinations (month)***		10.77 ± 9.74	17.00 ± 17.61	0.192
***Maximum dosage of RT for temporal lobes (Gy)***				
Right	NA	66.14 ± 8.31^#^	66.54 ± 9.06*	0.911
Left	NA	69.49 ± 5.87^#^	64.41 ± 7.71*	0.073
***The location of RE***				
Right, n	NA	NA	3 (16.7)	NA
Left, n	NA	NA	6 (33.3)	
Bilateral, n	NA	NA	9 (50.0)	

NA denotes not available. #Denotes radiation dose of three patients were not available. *Denotes radiation dose of 10 patients were not available.

### Magnetic Resonance Imaging Acquisition

Imaging data were acquired on a Magnetom Tim Trio 3T scanner (Siemens, Munich, Germany) using blood oxygen level-dependent (BOLD) and three-dimensional T1-weighted magnetization prepared rapid acquisition gradient echo (MPRAGE) sequences. Scanning parameters for BOLD and three-dimensional (3D) T1 MPRAGE sequences are documented in our previous studies (Zhang et al., 2018, 2020a; 2021). Briefly, for the BOLD sequence, the parameters were as follows: repetition time (TR) = 2,400 ms, echo time (TE) = 30 ms, field of view (FOV) = 230 mm × 230 mm, matrix size = 64 × 64, time points = 240, flip angle = 90°, and 40 axial slices. Patients were instructed to remain calm and awake with eyes closed during the resting-state fMRI scanning. For the 3D T1 MPRAGE sequence, the parameters were as follows: voxel size = 1.0 mm × 1.0 mm × 1.0 mm, FOV = 256 mm × 256 mm, matrix size = 256 × 256, thickness/gap = 1.0/0 mm, TR = 2,300 ms, TE = 2.98 ms, flip angle = 9°, and 176 sagittal slices.

### Functional Magnetic Resonance Imaging and Structural Magnetic Resonance Imaging Data Preprocessing

The fMRI and structural MRI data were preprocessed using Data Processing Assistant for Resting-State fMRI Advanced Edition^1^, the VBM8^2^ modules in the toolbox for Data Processing and Analysis for Brain Imaging (DPABI)^3^, and the SPM12^4^ software implemented in MATLAB 2016b.

The main preprocessing steps for BOLD fMRI data and 3D T1 data were described in Supplementary Appendix.

### Regional Homogeneity Mapping Acquisition and Regional Homogeneity/Voxel-Based Morphometry Calculation

The preprocessed BOLD data were used to calculate ReHo by measuring the similarity between the time series in a given voxel and those in the 26 neighboring voxels (Zang et al., 2004). Individual ReHo maps were obtained for each participant for subsequent analyses (Zhang et al., 2020a).

As both the ReHo maps and the modulated GM maps were normalized to the MNI coordinate system, we performed voxel-wise ReHo/VBM analysis by calculating the ratio of the ReHo value to GM volume of the voxel at the same coordinate. Fisher’s z-transformation was used to improve the normality of the obtained ReHo/VBM measurements. The z-transformed ReHo/VBM maps were smoothed using a 6-mm full-width-at-half-maximum isotropic Gaussian kernel and used for subsequent intergroup statistical and classification analyses.

### Support Vector Machine Analysis

The easylearn software (Pedregosa et al., 2011)^5^ was used to perform the machine learning analysis. A linear kernel support vector machine (SVM) classifier was used for the classification of RE. Detailed information on this process is described below.

#### Cross-Validation

We conducted a nested stratified K-fold (*K* = 10) cross-validation (CV) setup. The inner CV (first CV) served to optimize regularization parameters, while the outer CV (second CV) estimated predictive performance when using optimal parameters obtained in the inner CV. In each fold, the algorithm splits the sample into 10 equal parts, of which one part is used as a test set to estimate the performance of a model (outer CV) that is trained by the training set (the remaining nine parts), which used inner CV for hyperparameter tuning. The procedure was repeated until every part was used as the test set.

#### Feature Selection and Model Construction

Feature selection was strictly limited to the training set. During each fold of inner CV, two-sample *t*-tests between the Post-RT RE_+ve_ and Post-RT-RE_–ve_ groups were performed with a value of *p* < 0.05 to obtain a mask for the subsequent feature dimension reduction analysis. The least absolute shrinkage and selection operator (LASSO) was then used to select important features for improving classification efficiency. The selected features were then trained to build a classification model. The optimal classification model selected by the inner CV was tested on the test set. Classification accuracy, sensitivity, specificity, and area under the ROC curve (AUC) were calculated for performance evaluation. The classifier performance was accessed using a permutation test (repeated 1,000 times).

### Statistical Analysis

#### Clinical Data Analysis

Several statistics were used to describe different data types. Frequencies were used to describe qualitative data. Means and standard deviations were used to describe quantitative clinical data with a Gaussian distribution, and medians and interquartile ranges were used to describe non-normally distributed data. A chi-square test was used to examine intergroup differences in clinical stage, sex, and RT technique, and a one-way analysis of variance was used to detect intergroup differences in age. For the post-RT subgroups, a two-sample *t*-test was used to examine intergroup differences in maximum temporal lobe RT dosage and the time interval between RT and fMRI examination. For all analyses, *p* < 0.05 was considered significant.

#### Regional Homogeneity/Voxel-Based Morphometry Analysis

To evaluate intergroup differences in the structure–function coupling, all voxel-wise contrasts of the smoothed ReHo/VBM maps were performed using the statistical analysis module in the DPABI toolbox (see text footnote 3). Specifically, a two-sample *t*-test was applied to examine intergroup differences in ReHo/VBM using pairwise comparisons, with sex and age considered nuisance covariates. We used a two-tailed false-discovery rate (FDR) correction (*p* < 0.01) for multiple intergroup comparisons. In order to uncover the possible inter-group differences between the two subgroups (post-RT-RE_+ve_ and post-RT-RE_–ve_) and reveal the candidate neural mechanism of structure–function decoupling in RBI, alphasim correction with a threshold of *p* < 0.001 at the voxel level and *p* < 0.01 at the cluster level was used if the intergroup comparison results could not be corrected using the FDR method. To examine the relationship between ReHo/VBM alterations and radiation dose to the ipsilateral temporal lobe, we performed a voxel-wise Pearson’s correlation between the ReHo/VBM value and the maximum radiation dose administration confined to the bilateral temporal lobes (*p* < 0.05, FDR corrected).

## Results

### Clinical Data

A total of 62 patients with NPC were included, comprising 48 male and 14 female patients. Age ranged from 14 to 63 years, with a mean age of 44.27 years. The tumor stage ranged from T1N0M0 to T4N2M0. No significant differences in age (*p* = 0.867), sex (*p* = 0.927), or clinical stage (*p* = 0.592) were observed among the pre-RT, post-RT-RE_–ve_, and post-RT-RE_+ve_ groups. In the post-RT group, we found no significant differences between the post-RT-RE_–ve_ and post-RT-RE_+ve_ subgroups in the time interval between RT and fMRI examination (*p* = 0.192), RT technique (*p* = 0.138), or maximum dosage of RT (MDRT) to the right (*p* = 0.911) and left (*p* = 0.073) temporal lobes. In the post-RT-RE_+ve_ group, brain necrotic lesions in the left, right, and bilateral temporal lobes were detected in six, three, and nine patients, respectively (Table 1).

### Regional Homogeneity/Voxel-Based Morphometry Coupling Analysis

**FIGURE 2:**
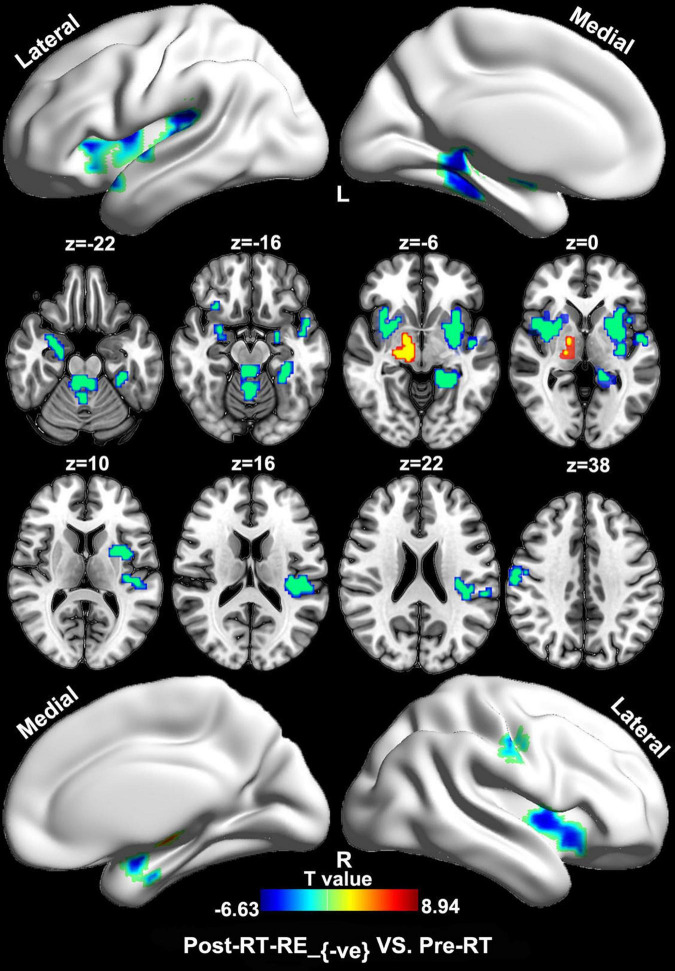
Between-group differences in ReHo/VBM (pre-RT vs. post-RT-RE_–ve_). Compared with the pre-RT group, patients in the post-RT-RE_–ve_ group showed increased ReHo/VBM coupling values in SN and decreased values in brainstem, the cerebellum anterior lobe and vermis, the bilateral hippocampus/parahippocampal gyrus, the bilateral insula, and the right precentral as well as the postcentral gyrus (*p* < 0.05, FDR corrected).

**FIGURE 3:**
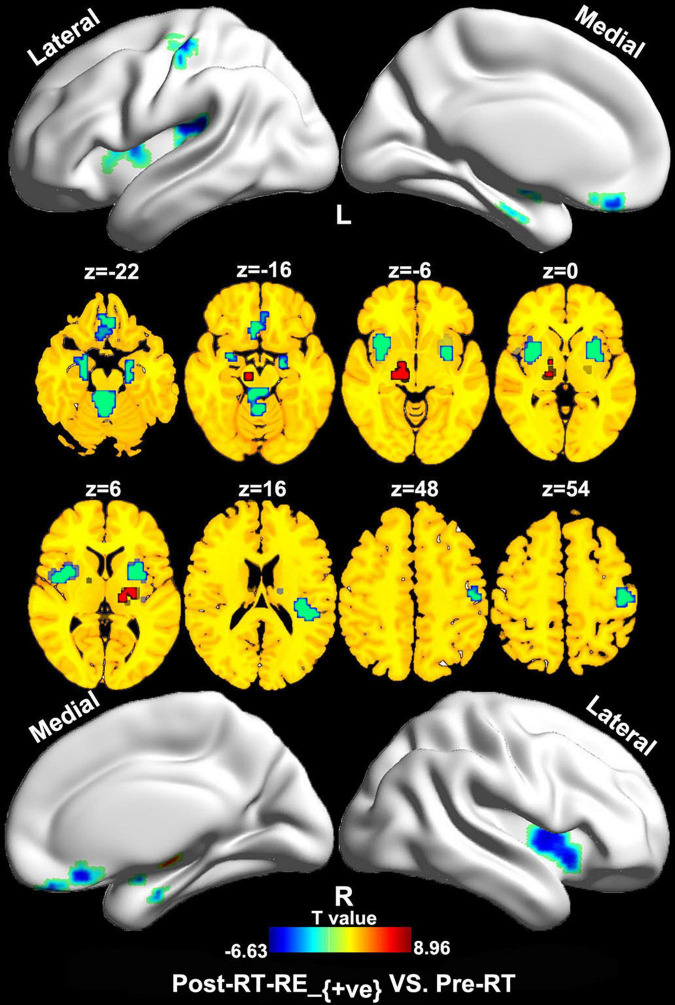
Between-group differences in ReHo/VBM (pre-RT vs. post-RT-RE_+ve_). Compared with the pre-RT group, patients in the post-RT-RE_+ve_ group displayed decreased ReHo/VBM coupling values in the cerebellum anterior lobe and vermis, the bilateral MTL, the midbrain tegmentum, the MPFC, the bilateral insula, the left inferior parietal lobule, and the right precentral and postcentral gyrus (*p* < 0.05, FDR corrected). Meanwhile, these patients also showed increased ReHo/VBM coupling values in the right SN, the bilateral thalamus, and the putamen (*p* < 0.05, FDR corrected).

Compared with the pre-RT group, patients in the post-RT-RE_–ve_ group showed higher ReHo/VBM coupling values in the substantia nigra (SN) and lower values in the brain stem (e.g., midbrain tegmentum and pons), the anterior lobe and vermis of the cerebellum, the bilateral medial temporal lobes (MTLs; hippocampus/parahippocampal gyrus), the bilateral insula, and the right precentral and postcentral gyri (*p* < 0.05, FDR corrected; Figure 2). Compared with the pre-RT group, patients in the post-RT-RE_+ve_ group displayed lower ReHo/VBM coupling values in the anterior lobe and vermis of the cerebellum, the bilateral MTL, midbrain tegmentum, the medial prefrontal cortex (MPFC), the bilateral insula, the left IPL, and the right precentral and postcentral gyri (*p* < 0.01, FDR corrected; Figure 3) and higher ReHo/VBM coupling values in the right SN and the bilateral thalamus and putamen (*p* < 0.01, FDR corrected; Figure 3). Compared with the post-RT-RE_–ve_ group, patients in the post-RT-RE_+ve_ group showed significantly higher ReHo/VBM coupling values in the right superior temporal gyrus (STG; *p* < 0.001, alphasim corrected; Supplementary Figure 1).

### Correlation Analysis

In the post-RT group (which includes the post-RT-RE_–ve_ and post-RT-RE_+ve_ subgroups), significant negative correlations were observed between MDRT to the ipsilateral temporal lobe and ReHo/VBM coupling values in the ipsilateral middle temporal gyri (MTGs; Supplementary Figure 2).

### Support Vector Machine Analysis

**FIGURE 4:**
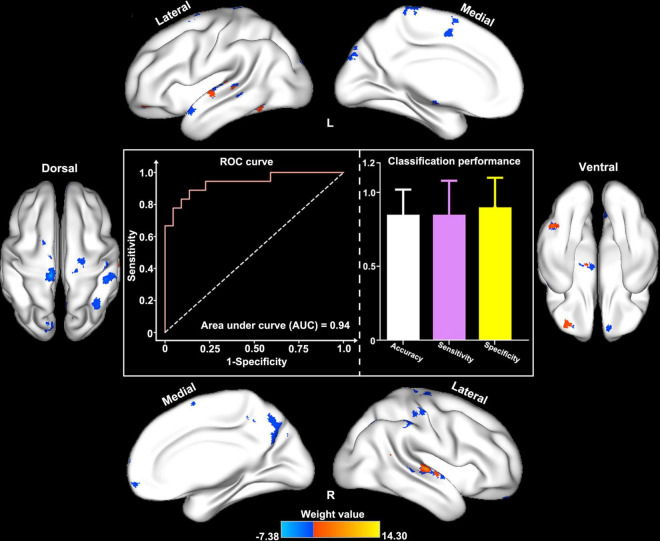
Classification performance of structure–function decoupling in the prediction of RE. ReHo/VBM coupling values in a series of cortical brain regions (e.g., the bilateral STG and MTG, the bilateral pre/postcentral gyri, the paracentral lobules, the right precuneus and inferior parietal lobule, the right MPFC, and the left SN) differentiated patients with and without RE with the area under the curve, accuracy, sensitivity, and specificity of 0.94, 88.0, 85.0, and 90.0%, respectively.

In the classification model construction, we observed that ReHo/VBM coupling values in several cortical brain regions (e.g., the bilateral STG, the bilateral precentral and postcentral gyri, the paracentral lobules, the right precuneus and IPL, the right MPFC, and the left SN) differentiated patients with and without RE, with an AUC, accuracy, sensitivity, and specificity of 0.92, 85.1, 85.8, and 85.7% in the training set; and 0.94, 88.0, 85.0, and 90.0% in the test set, respectively (Figure 4).

## Discussion

This is the first study using ReHo/VBM to detect the effects of RT on structure–function coupling in normal-appearing GM of patients with NPC. Several brain regions with structure–function decoupling were observed within and outside the radiation field, which suggested that RBI is a multisystem disease that involves the dysfunction of regional structure and functional coordination in both near-end (nearby or in the path of irradiation) and far-end brain regions. Our finding of a negative correlation between MDRT to the ipsilateral temporal lobe and ReHo/VBM value in the ipsilateral MTG indicated that the alteration in brain structure–function coupling was primarily induced by RT. Notably, we found the structure–function decoupling in brain regions constituting the sensorimotor system, and the default mode network (DMN) exhibited impressive classification performance in the proposed model, which indicated that structure–function decoupling in these functional systems plays a crucial role in the identification of RE.

In this study, a ReHo/VBM ratio was adopted to characterize the coupling between the functional response and GM volume in the brain. For an individual, the voxel-wise ReHo/VBM ratio measures the amount of regional functional demand per unit of regional GM morphological changes, which could reflect the structure–function coupling for a specific voxel or region (Zhu et al., 2017). Therefore, the voxel-wise ReHo/VBM ratio could be used to identify alterations in the structure–function coupling in patients with NPC, followed by RT, which cannot be obtained by the separate ReHo or VBM analysis.

Compared with the pre-RT group, patients in the post-RT group (e.g., post-RT-RE_–ve_ and post-RT-RE_+ve_ subgroups) showed lower ReHo/VBM values in the bilateral MTL, the midbrain tegmentum, and the bilateral insula. The MTLs are responsible for learning and memory consolidation (Wittenberg and Tsien, 2002; Squire et al., 2004). Thus, structure–function decoupling in this area would likely lead to cognitive impairment, which is supported by a recent report of radiation-induced poor short-term memory in patients with NPC (Hsiao et al., 2010). The midbrain tegmentum plays an important role in visceral (e.g., cardiovascular control) and movement functions, sleeping rhythm, and pain modulation (Caminero and Cascella, 2021). Indeed, radiation-induced cardiovascular autonomic impairment (as evidenced by lower cardiovascular autonomic parameters and scores) and sleep disturbances were well-documented in patients with NPC during RT in previous studies (Huang et al., 2013; Qin et al., 2015). It is tempting to speculate that our findings of disrupted structure–function coupling in the midbrain may be central adaptive alterations secondary to the impaired visceral function and sleeping rhythm. Our findings of structure–function decoupling in the bilateral insula and midbrain tegmentum [covering the periaqueductal gray matter (PAG)] may be related to the abnormal encoding of pain intensity, impaired inhibition of nociceptive inputs, and augmented pain perception (Ossipov et al., 2014; Watson, 2016; Zhang et al., 2020b). In fact, chronic headache and oral and neuropathic pain are common clinical symptoms in patients with RT-induced injury (Gu et al., 2014; Hu et al., 2018), and we speculate that our findings of altered ReHo/VBM in the insula and PAG may be a central response to RT-induced peripheral nociceptive stimuli. Unfortunately, the detailed data related to cognition, autonomic nervous system (ANS) function, sleep quality, as well as pain rating were not available for the patients with NPC in this study. The relationship between altered ReHo/VBM and the evaluation of data in cognition, ANS, sleep, and pain in patients with NPC after RT should be explored in future studies.

Compared with the pre-RT group, patients in the post-RT group showed significantly lower ReHo/VBM values in the precentral and postcentral gyri, the cerebellum, and the brainstem and significantly higher ReHo/VBM values in the SN, the putamen, and the thalamus. The observed structure–function decoupling in the precentral and postcentral gyri, the cerebellum, and the brainstem is consistent with previous neuroimaging studies that showed increased ReHo in the postcentral gyrus, decreased ReHo and cortical thickness in the precentral and postcentral gyri and the brainstem, and impaired functional connections between the cerebellum and the sensorimotor network (Ma et al., 2016; Lin et al., 2017; Zhang et al., 2020a), which indicates the impairment of sensorimotor function. Anatomically, the precentral and postcentral gyri are the highest regulation centers of the descending motor system and ascending somatosensory pathway, with extensive cortical–brainstem–cerebellum connections *via* ascending and descending projections (Lin et al., 2017; Zhang et al., 2021). During RT of NPC, the cerebellum and the brainstem are included in the radiation field and are exposed to a high dose of radiation. We speculate that the structure–function decoupling in the sensorimotor system may be secondary to radiation-related brainstem and cerebellum injury. In contrast to the decreased changing pattern of ReHo/VBM values in the pre-/post-central gyri and the cerebellum, our finding of increased ReHo/VBM values, followed by RT, in the SN, the putamen, and the thalamus is of particular interest. Considering that the SN-striatum-thalamus (SST) pathway and the descending motor system function together physiologically to perform a voluntary movement (Davis et al., 2015; Zhang et al., 2020b), it is tempting to speculate that the enhanced structure–function coupling in the SST pathway compensates for the impaired motor function caused by the aforementioned structure–function decoupling in the primary motor cortex. The SN, the putamen, and the thalamus are interconnected through the facilitation or inhibition of dopaminergic neurotransmitter release to perform a multitude of functions, such as motor planning and rewarding processing (Davis et al., 2015; Selemon and Begovic, 2020). Recent animal studies have reported that dopaminergic neurons are easily damaged by RT *via* direct particle strikes, oxidative stress, and microglial activation (Joseph et al., 2000; Rabin et al., 2004; Koike et al., 2005; Davis et al., 2015). As such, to maintain motor function as much as possible in patients with NPC, the increasing functional need of the SST pathway for voluntary movement and the RT-induced decrease in dopaminergic neurons may be two important factors that contribute to enhanced structure–function coupling in the SST pathway. However, further investigations on the sensorimotor system and the SST pathway with neuroimaging data, detailed neuro-electrophysiological data, and behavior tests are required to confirm this notion.

During the process of SVM model construction, ReHo/VBM values in several brain regions, such as the precentral and postcentral gyri, the precuneus, the IPL, and the MPFC, showed an excellent classification performance, which indicated that structure–function coupling alterations in sensorimotor system and DMN-related brain regions play an important role in identifying patients who are likely to develop RE. These findings are consistent with recent functional MRI study findings (Zhang et al., 2021; Zhao et al., 2021), which indicated that the biological classification information obtained from the sensorimotor system, and DMN was stable and robust. Moreover, ReHo/VBM values in regions that receive dense radiation dose exposure (e.g., the MTL) contributed less to the prediction of RE. These findings suggest that RT-induced structure–function coupling alterations in the inferior part of the temporal lobes are relatively small and can be suppressed by more powerful alterations in the abovementioned two brain networks. However, the exact cause of such an unexpected finding is unclear. Off-target effects of RT on near-end brain regions (e.g., the MTL and the brainstem) that alter brain activity in far-end regions (e.g., the precentral and postcentral gyri) may be one explanation (Beera et al., 2018; Zhang et al., 2021). Our finding of impressive classification performance of structure–function coupling in the precentral and postcentral gyri, the IPL, and the MPFC suggests that more attention should be paid in future studies to the functional and/or structural alterations in far-end brain regions rather than those within the radiation fields.

Several limitations of this study should be addressed. First, although potential confounding factors, such as age, RT technique, TNM stage, chemotherapy regimen, and the main side of the nasopharyngeal tumor, were balanced across groups, our findings should be interpreted with caution because of the cross-sectional study design. These confounding factors should be controlled in future longitudinal studies. Second, the lack of histopathological confirmation of RE is another limitation of this study; however, brain biopsies of RE are currently not possible in clinical practice because of ethical reasons and non-negligible medical risks, such as cerebral hemorrhage. Third, detailed information regarding RT-induced alterations in psychological state, cognitive, auditory, and sensorimotor functions, pain rating, ANS function, sleep quality, and quality of life were not acquired, which weakens the interpretability of our findings. Fourth, some brain regions observed in this study were discussed together at the brain network level, such as the sensorimotor system, DMN, and SST pathway; however, these speculations should be validated by further brain network-based research and specific neural pathway studies. Fifth, as the time interval between RT and fMRI examinations varied, further studies with a series of RT time points are warranted to obtain the dynamic profile of radiation-induced structure–function coupling alterations in patients with NPC. Sixth, the lack of genetic and high-field (e.g., 7.0 T) MRI data limits the precision and depth of our study, and future imaging genetic studies with high-resolution MRI data and detailed genetic information were needed to uncover the genetic basis and candidate neural mechanism underlying the radiation-induced laminar functional and structural alterations. In addition, the small sample size in this study may have lowered the statistical power, which may have resulted in overfitting, although we minimized this issue by using multiple comparison corrections and building an SVM model.

An independent dataset would provide a better/reliable metric of classifier performance, as compared to the nested stratified cross validation approach. This could be part of future studies etc.

## Conclusion

Radiation-induced alterations in ReHo/VBM were observed in multiple brain regions that involved the bilateral MTL, the midbrain tegmentum, the bilateral insula, the sensorimotor network, and the SST pathway. Structure–function coupling alterations in the precentral and postcentral gyri and the DMN-related regions exhibited excellent performance in identifying patients who were likely to develop RE. These findings suggest that ReHo/VBM may be a novel and effective imaging metric that reflects the neural mechanism underlying RBI in patients with NPC.

## Data Availability Statement

The original contributions presented in this study are included in the article/Supplementary Material, further inquiries can be directed to the corresponding author.

## Ethics Statement

The studies involving human participants were reviewed and approved by the Medical Research Ethics Committee of Xiangya Hospital, Central South University. The patients/participants provided their written informed consent to participate in this study.

## Author Contributions

Y-mZ, LL, Y-fK, and J-mG conceived and designed the experiments. Y-fK, Y-mZ, and HD analyzed the data. Y-mZ, LL, J-mG, Y-fK, L-zL, and R-tC contributed to materials and analysis tools. Y-mZ, Y-fK, R-tC, and HD wrote the manuscript. All authors read and approved the final manuscript.

## Conflict of Interest

The authors declare that the research was conducted in the absence of any commercial or financial relationships that could be construed as a potential conflict of interest.

## Publisher’s Note

All claims expressed in this article are solely those of the authors and do not necessarily represent those of their affiliated organizations, or those of the publisher, the editors and the reviewers. Any product that may be evaluated in this article, or claim that may be made by its manufacturer, is not guaranteed or endorsed by the publisher.

## Footnotes

1
http://rfmri.org/DPARSF_V2.3

2
http://www.neuro.uni-jena.de/vbm/download/

3
http://rfmri.org/dpabi

4
https://www.fil.ion.ucl.ac.uk/spm/software/spm12/

5
https://github.com/lichao312214129/easylearn

## Abbreviations

NPC: nasopharyngeal carcinoma
RT: radiotherapy
ReHo: regional homogeneity
VBM: voxel-based morphometry
MDRT: maximum dosage of RT
RBI: radiation-induced brain injury
fMRI: functional magnetic resonance imaging
GM: gray matter
FC: functional connectivity
RE: radiation encephalopathy
UICC/AJCC: International Union against Cancer/American Joint Committee on Cancer
TNM: tumor nodes and metastasis tumor staging system
IMRT: intensity-modulated radiation therapy
2D-CRT: two-dimensional conventional radiotherapy
BOLD: blood oxygen level-dependent
3D MPRAGE: three-dimensional T1-weighted magnetization prepared rapid acquisition gradient echo
TR: repetition time
TE: echo time
FOV: field of view
DPABI: Data Processing and Analysis for Brain Imaging
MNI: Montreal Neurological Institute
CSF: cerebrospinal fluid
SVM: support vector machine
CV: cross-validation
LASSO: the least absolute shrinkage and selection operator
ROC: receiver operator characteristic curve
FDR: false-discovery rate
SN: substantia nigra
MTL: medial temporal lobes
MPFC: medial prefrontal cortex
MTG: middle temporal gyri
STG: superior temporal gyrus
IPL: inferior parietal lobule
DMN: default mode network
PAG: periaqueductal gray matter
SST: SN-striatum-thalamus.
