# Supplementary material for: Structure–Function Decoupling: A Novel Perspective for Understanding the Radiation-Induced Brain Injury in Patients With Nasopharyngeal Carcinoma
Source: Front Neurosci. 2022 Jul 4;16:915164. doi: 10.3389/fnins.2022.915164 (PMC9289669; doi:10.3389/fnins.2022.915164)
Supplement: Supplementary file 1 [file Presentation_1.pdf]

Appendix

FMRI and structural MRI data preprocessing

The main preprocessing steps for BOLD fMRI data were performed as follows: 1) the first 10 volumes of each scan were discarded; 2) slice timing and head motion correction was performed; 3) images were spatially normalized to the Montreal Neurological Institute (MNI) template; 4) nuisance signals (including head motion parameters, cerebrospinal fluid [CSF], WM, and global mean signal) were regressed out; 5) data were temporal band-pass filtered (0.01–0.1 Hz). Because rsfMRI data are sensitive to head motion (Van Dijk et al. 2012, Yan et al. 2013), subjects were excluded if their maximum rotation was > 3.0° or their maximum translational displacement (x, y, or z directions) was > 3.0 mm.

The main preprocessing steps for the 3D T1 data were performed as follows: all high-resolution structural images were segmented into GM, WM, and CSF. Using the high-dimensional DARTEL normalization model (Ashburner 2007), the GM images were normalized to MNI space. Images were then modulated to ensure that actual GM volumes were well preserved following spatial normalization (Peng et al. 2020). The modulated GM maps were then resampled to a voxel size of 3 × 3 × 3 mm for subsequent analyses.

Results

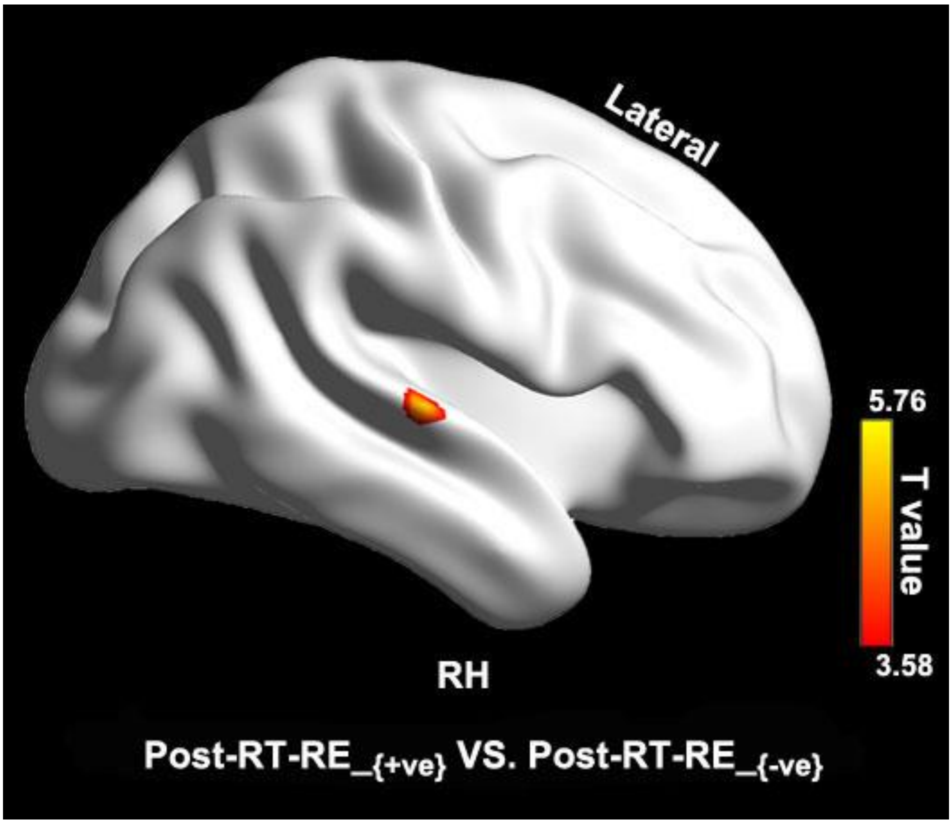

FIG S1. Between-group differences in ReHo/VBM (Post-RT-RE_$_{\{-ve\}}$ vs. Post-RT-RE_$_{\{+ve\}}$). Compared with Post-RT $_{\text{non-RE}}$ group, patients in the Post-RT $_{\text{RE proved in follow-up}}$ group showed a significantly increased ReHo/VBM coupling values in the right superior temporal gyrus ($P < 0.05$, Alphasim correction).

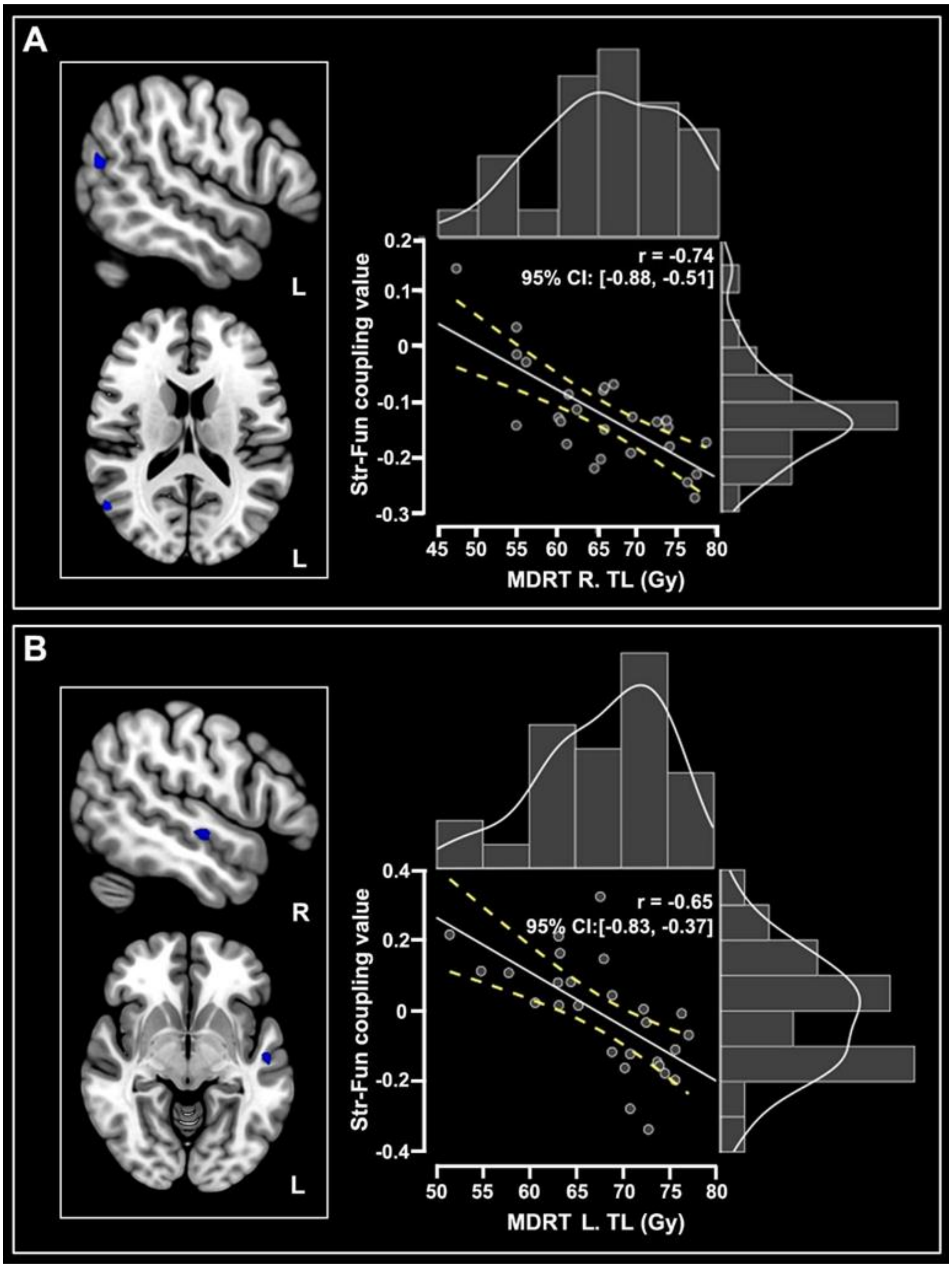

FIG S2. Correlations between MDRT and ReHo/VBM. In the Post-RT patients group, a significant negative correlation was observed between ReHo/VBM of the right middle temporal gyrus (MTG) and the ipsilateral MDRT (A). A significant negative correlation was observed between ReHo/VBM of the left MTG and ipsilateral MDRT (B).

References

Ashburner, J. (2007). A fast diffeomorphic image registration algorithm. *Neuroimage* 38, 95-113.doi:10.1016/j.neuroimage.2007.07.007

Peng, Y., Zhang, Y., Chen, Z., Peng, H., Wan, N., Zhang, J., et al. (2020). Association of serum neurofilament light and disease severity in patients with spinocerebellar ataxia type 3. *Neurology* 95, e2977-e2987.doi:10.1212/WNL.0000000000010671

Van Dijk, K. R., Sabuncu, M. R. and Buckner, R. L. (2012). The influence of head motion on intrinsic functional connectivity MRI. *Neuroimage* 59, 431-438.doi:10.1016/j.neuroimage.2011.07.044

Yan, C. G., Cheung, B., Kelly, C., Colcombe, S., Craddock, R. C., Di Martino, A., et al. (2013). A comprehensive assessment of regional variation in the impact of head micromovements on functional connectomics. *Neuroimage* 76, 183-201.doi:10.1016/j.neuroimage.2013.03.004
